# Is coconut water truly a miracle drink in pregnancy or a myth?

**DOI:** 10.12669/pjms.40.8.8817

**Published:** 2024-09

**Authors:** Maryam Raza

The benefits of coconut water have transcended from generation to generation in the subcontinent with each glorifying its miraculous properties for all pregnancy-related issues. But to what extent can we validate that scientifically?

Cocos nucifera L or Coconut Water (CW) contains glucose, protein, numerous inorganic ions (Mg, potassium, sodium etc.), sugar alcohols (Myo-inositol, Sorbitol etc.), and even essential amino acids and fatty acids, all of which contribute significantly to its use as a natural alternative for oral rehydration and in some remote areas it has even been used intravenously.^1^ CW has the quickest recovery rehydration index to other drinking supplements due to it having similar electrolyte composition, specific gravity and osmolarity to blood.^2^ Research has also shown to have beneficial anti-hypertensive and anti-diabetic properties which can be considered for pregnant patients.^1^

Pregnancy comes with its own new onset of challenges such as nausea and vomiting in the first trimester. The go-to treatment is usually supplementation with vitamin B6. Since the first trimester is a very sensitive period in which the fetus’s organogenesis occurs, it is recommended to prescribe as natural of a remedy as possible. A recent RCT conducted in India showed that a daily dose of 300ml of coconut water significantly helped reduce symptoms of morning sickness.^3^

In the third trimester, one of the complications that can occur is amniotic fluid stained by meconium. It is associated with high rates of emergency C-sections, meconium aspiration syndrome and a low Apgar score in the newborn. In the city of Palangkaraya, Indonesia pregnant woman have a habit of daily consuming CW as it is culturally correlated with preventing meconium staining of amniotic fluid. The use of coconut water arises due to similarities in its composition to blood allowing quick absorption of electrolytes, mainly potassium.^2^ In a study conducted by Ariestini et al.,^2^ it was theorized that maternal salivary potassium levels have a direct effect on increasing newborn salivary potassium levels and hence a negative impact on amniotic fluid meconium level. The results of the study showed a significant decrease in meconium staining of amniotic fluid.^2^

Lastly, the benefits of CW were also studied during the process of labor by Wardhani et al.^4^ as prolonged labor can increase the incidence of C-sections and further complications. In the study patients in the latent phase of labor were given 500ml of coconut water to assess progress in the active phase. In comparison to the control group results showed a mean increase in the opening of childbirth by 54 minutes thus showing a positive correlation.^4^

It can be concluded that though few aspects of culture and tradition regard CW to unreasonable standards, it can be said that some truth does exist scientifically.
